# 

**Published:** 1865-04

**Authors:** 


### CHICAGO MEDICAL JOURNAL.

**Terms, $2.00 per Annum.**

### CONTENTS OF THE APRIL NUMBER.

PAGE.

ORIGINAL COMMUNICATIONS.

Diptheria. By James S. Whitmire, M. D., of Metamora, Ill.............. 145

Excision of the Tonsil. By John T. Meyers, M. D., Fairmount, Ill...... 153

On the Therapeutic Value of Nux Vomica in the Treatment of Asthenic Forms of Disease. By A. Middleditch, M. D., Waterloo, Iowa...... 154

How to Preserve Vaccine Matter. By Dr. D. Prince, of Jacksonville, Ill. 156

Report of an Unusual Case. By Dr. G. Meachem, M. D., of Racine. Wisconsin.................................................. 157

Clinical Lectures on Diseases of the Eye. By E. L. Holmes, M. D., of Chicago, Lecturer on Diseases of the Eye and Ear, in Rush Med. Col., etc.—Choroiditis.............................................. 160

To the Medical Profession of Illinois........................................ 164

Chloroform. By M. M. Eaton, M. D., of Peoria, Ill........................ 165

SELECTED.

Physiology of Tasting. By M. Norton Folsom................................ 172

On the Mechanism of Speech. By Isaac Pidduck, M. D..................... 174

Protection from Small Pox by means of Vaccination and Re-Vaccination. 176

Book Notices and Reviews..................................................... 188

Editorial and Miscellaneous................................................... 191

